# On the Present State of Our Knowledge of Opium

**Published:** 1804-08-01

**Authors:** A. F. Gehlen

**Affiliations:** Berlin


### On the present State of our Knowledge of Opium.

*By* A. F. Gehlen, *of Berlin.*

( Continued from our last pp. 38—41. )

Mr. JOSSE likewise remarks, that the water with which opium is washed, and which contains the extractive matter in solution, becomes fatty, and that also the surface of the glutinous matter remaining on the filtrum is covered with a coloured fatty cuticle, which he takes to be merely accidental, as he thinks it may be derived from the oil or fat with which those persons who prepare the opium-cakes in Asia anoint their hands. This opinion, however, is by no means probable, particularly as the late observations of Derosne and Proust, have evinced that an oily matter is really contained in opium. Proust thinks it to originate from the pollen of papaver, thus partaking of the nature of wax. This conjecture is confirmed by Mr. Derosne's method of obtaining it. After having several times extracted the opium with water and then with alcohol in gentle heat, he let alcohol boil with the residuum, and filtrated the liquor, which on cooling deposited an oily solid black brown substance. This being again dissolved in boiling alcohol, it was also precipitated on cooling in a very divided form and with a yellowish grey colour. It is now known to be a property of wax, that it dissolves in boiling alcohol, but is again separated on cooling. Mr. Derosne obtained from one pound of opium about an ounce of this substance, which, as this gentleman says, imparts the particular smell to opium, and is the only constituent that retains it, while the other constituent particles entirely lose this odour. Hence it may be explained, why Mr. Josse could ascribe the smell and stupifying qualities of opium to what he calls gluten, as this oily substance could not be separated from it, according to his manner of proceeding.

Another substance, which has been likewise overlooked in some of the modern analyses, is a particular saline matter, already noticed by Neumann, Hoffmann, and Tralles, who thought it to be an acid; an opinion lately renewed by Mr. Proust. The observations however which Mr. Derosne has published on this subject are the following. He extracted opium with ten times its quantity of distilled water, which operation he repeated till all the parts soluble in water were separated, and he gently evaporated the liquors, which he had thus obtained, to the consistency of a thick syrup. On cooling, the thick liquor became granulated, which seemed to indicate the presence of a separated substance. On diluting it with four parts of water it appeared turbid, and a considerable sediment was separated on the bottom of the vessel. The liquor being decanted and again evaporated, still yielded a small portion of it. After having collected it on the filtrum and washed it, it showed a dark brown colour, and at the first sight seemed to be composed of resin and oxydated albuminous matter; but on examining it more closely, it appeared to consist of an innumerable quantity of small shining crystals. Boiling water drew out of it a small portion of extractive matter. Alcohol, when boiling, dissolved them, but on cooling deposited them again in a crystalline form. The same substance may be obtained by treating the residuum, which is not soluble in water, with alcohol.

Mr. Derosne having digested it with six parts of alcohol, in a heat of 35°—40° Reaum. filtrated the dark red liquor when still warm, which on cooling deposited crystals. By repeated solution of this substance in alcohol and crystallisation it is obtained quite pure and free from resin and the abovementioned oily matter. One pound of opium yields about 40 grammes French weight. In this state, purified by repeated crystallizations, opium appears white and crystallized in regular fasciles or prisms. It is without smell and odour, not soluble in cold water, but dissolves in 400 parts of boiling water, from which however it is again precipitated on cooling. Tincture of turnsol is not reddened by it. It requires of boiling alcohol about 24 parts for solution, but in the cold about 100 parts. One of the most distinguishing characters of this substance is its easy solubility in acids, vegetable as well as mineral, without the assistance of heat. On saturating such a solution with an alkali, that substance is precipitated in form of a white powder. It is made somewhat soluble in water by caustic alkalies; but it is precipitated from this solution by adding ng a small portion of an acid. It is dissolved by ether and volatile oils in the warmth, but separates again in a liquid oily form, and shoots soon after into crystals. Thrown on living coals it takes fire, and burns with the same flame as other combustible vegetable substances do. When heated in a spoon it gradually melts like small pieces of wax. When exposed in a retort to a gradually increased fire, it begins to melt and to puff up, whereby the retort is filled with white fumes which are condensed near the neck of the retort as an oily yellowish substance, and at the same time some phlegma with carbonat of ammonia passes over. Towards the end of the operation carbonic acid gas, dry ammonia, and carbonated hydrogen gas are disengaged, while there remains in the retort a voluminous, light, spongy, and shining coal. The oily substance deposed in the neck of the retort is very tough, and possesses a particular aromatic smell and an acrid taste. Nitric acid poured on the grossly pulverised crystals imparts to them a reddish colour, dissolving them afterwards with great ease. The solution, heated and evaporated, yields crystals of oxalic acid in a proportionably great quantity. The residuum of the solution is very bitter.

From the just mentioned properties of this substance, Mr. Derosne thinks himself entitled to consider it as a particular matter, and as a new proximate constituent of vegetables; and he remarks, that on account of those properties it can be no acid, as has been maintained by some chemists. The easy solubility of this substance, and likewise of the resinous and oily particles of opium in acids, may account for the action of vinegar, employed as an antidote of opium, and he believes that other acids operate in the same way, and are capable of preventing the deleterious effects of opium by dissolving those difficultly soluble parts of that substance. This opinion is contrary to that of Mr. Josse, who thinks acids to be very prejudicial to the body when opium has been given, as deleterious substances have a greater efficacy in a dissolved form; Mr. Derosne, however, assures us, that he has administered from six to eighteen grains of opium to dogs, which were all affected with the symptoms attending too great a dose of that substance, but most of them were relieved and cured by giving them vinegar.

Caustic and carbonated alkalis produce a copious precipitation in the aqueous solution of opium; Mr. Proust considers this precipitate as pure resin, but Mr. Derosne thinks it to be a compound substance. He added to a solution of opium in six parts of water, prepared in the cold, a solution of carbonat of kali, till nothing was precipitated. The remaining liquor, after being a little evaporated, yielded another small portion of that precipitate, which, when washed with cold water, had a greyish colour, a granulated appearance, and not much taste. Boiling alcohol dissolved about three-fourths of it, and received a dark red colour; and the solution being filtrated, yielded on cooling, an irregular reddish crystallization. The remaining alcohol contained another small portion, which could be separated by evaporation. The part which had been insoluble in alcohol was dissolved in boiling water; the solution had a dark colour, but on cooling a white powder fell to the bottom, which after being edulcorated and dried, was insoluble in boiling water, but thrown into a red hot crucible burned with a variously coloured flame, leaving behind carbonat of lime with a little kali. The same result was observed in that part which remained insoluble in water, on being dried and burnt. The part of the precipitate produced by alkali, which was insoluble in alcohol, consists consequently of lime, and a vegetable matter. The crystalline substance separated from the alcohol was the same which I have above mentioned, though it showed some difference, arising from the different manner of obtaining it. Its taste is a little bitter; it does not crystallize so regularly, and it seems to be more soluble; its solution changes the blue colour of the syrupus violarum into green. Being heated it crackles before it melts. Its solution in alcohol is not rendered turbid by the addition of water, but some time after small crystals appear in the liquor. The dry distillation yields the same results as have been above mentioned, except the remaining coal being less voluminous, and containing after the incineration more alkali, which seems to account for the little differences observed between this and the foregoing substance.

The aqueous solution of opium reddens the tincture of turnsol, according to Mr. Derosne, thus indicating the presence of an acid. He thought to discover the latter in the alkaline liquor, standing over the precipitate produced by alkali, on which account he evaporated the liquor to the consistency of a syrup, and suffered it to stand for several days, in order to produce a crystallization. Some time after crystals were found to be formed in the liquor, but they were few, and so combined with the extractive matter, that Mr. Derosne could not examine them.

On

On the whole, he thinks that the acid present in opium is nothing else but acetic acid, which is so frequently met with in other vegetable extracts. Besides those crystalline substances, the watery extract of opium contains, according to Mr. Derosne's researches, extractive matter and resin, which last is contained in it in a greater proportion, when the solution is made more concentrated, because the extractive matter renders the resin soluble. The latter however separates from the watery solution, by evaporation, to the consistency of a syrup, and by dissolving the extract again in form of a soft tough mass, which afterwards hardens. It may be perfectly separated by a proper treatment with alcohol, whereby part of it remains insoluble, which Mr. Derosne takes for oxydated extractive matter, and Mr. Buchholz for a close combination of resin with gluten. Lastly, the watery solution of opium contains some sulphat of lime and sulphat of kali, which may be separated by alcohol or by the incineration of the extract. The residuum of opium, which has been extracted with water and alcohol, consists of vegetables, frequently mixed with sand and small stones, from which, according to Mr. Derosne, a small portion of amylum, of mucilage and gluten may be extracted by boiling water. Mr. Buchholz obtained from the residuum, kaoutshouc, by treating it with ether.

From all those experiments and observations respecting the nature of opium, the following results may be drawn.

1. It is not yet sufficiently known in what manner opium is prepared in Asia, nor is it probable that common opium is the juice extilled from the poppy heads, inspissated in the air; as, according to Mr. Dubuc's observations, the juice spontaneously extilling from the capsules is not possessed of the narcotic smell, which common opium always possesses. The observations of Dubuc, and of Kuhn, lead us to conclude, that the opium extilling from the incisions of the capsules is kneaded and mixed with the fermented mass of squeezed poppy heads and leaves, and afterwards wrapt in the leaves of the plant.

2. Opium is a very compound substance. Besides the volatile narcotic principle, it contains *extractive matter, mucilage, resin,* and, according to several observations, *an oily matter,* resembling wax, to which the narcotic and deleterious properties of opium are by some particularly ascribed; farther, *a crystalline substance,* which is neither of a saline nor an acid nature; *a substance* similar to the albuminous matter which is separated from green vegetable ble juices, and which some consider as *gluten;* a small proportion of *kaoutshouc,* and the remainder of different vegetable particles. The proportion of these constituents seems not always to be the same; and from want of comparative examinations, it is not as yet ascertained whether all sorts of opium contain the same constituents, some of which undoubtedly require farther examination.

